# A New Composite Slab Using Crushed Waste Tires as Fine Aggregate in Self-Compacting Lightweight Aggregate Concrete

**DOI:** 10.3390/ma13112551

**Published:** 2020-06-03

**Authors:** Jing Lv, Tianhua Zhou, Hanheng Wu, Liurui Sang, Zuoqian He, Gen Li, Kaikai Li

**Affiliations:** School of Civil Engineering, Chang’an University, Xi’an 710061, China; zhouth@chd.edu.cn (T.Z.); wuhanheng@chd.edu.cn (H.W.); sangliurui@chd.edu.cn (L.S.); 2016128051@chd.edu.cn (Z.H.); 2017128067@chd.edu.cn (G.L.); 2017128062@chd.edu.cn (K.L.)

**Keywords:** self-compacting rubber lightweight aggregate concrete, composite slabs, flexural behavior, experimental study, finite element analysis, modified calculated method

## Abstract

A composite slab comprised of self-compacting rubber lightweight aggregate concrete (SCRLC) and profiled steel sheeting is a new type of structural element with a series of superior properties. This paper presents an experimental research and finite element analysis (FEA) of the flexural behavior of composite slabs consisting of SCRLC to develop a new floor system. Four composite slabs specimens with different shear spans (450 mm and 800 mm) and SCRLC (0% and 30% in rubber particles substitution ratio) are prepared, and the flexural properties including failure modes, deflection at mid-span, profiled steel sheeting, and concrete surface stain at mid-span and end slippage are investigated by four-point bending tests. The experimental results indicate that applying SCRLC30 in composites slabs will improve the anti-cracking ability under the loading of composite slabs compared with composite slabs consisting of self-compacting lightweight aggregate concrete (SCLC). FEM on the flexural properties of SCRLC composites slabs show that the yield load, ultimate load, and deflection corresponding to the yield load and the ultimate load of composite slabs drop as the rubber particles content increases in SCRLC. The variation of SCRLC strength has less impact on the flexural bearing capacity of corresponding composite slabs. Based on the traditional calculated method of the ultimate bending moment of normal concrete (NC) composite slabs, a modified calculated method for the ultimate bending moment of SCRLC composite slabs is proposed.

## 1. Introduction

The reclamation of waste tires is beneficial for saving resources and protecting the environment. As a result of the huge consumption of concrete, crushing waste tires into particles and using them as aggregate in concrete is an efficient way for reuse. In recent years, plenty of research studies have been conducted on the properties of rubber concrete [1,2,3,4,5]; moreover, applying rubber concrete in structural elements has also been attempted [6,7,8,9,10,11]. The results show that the utilization of rubber concrete in structural elements can make structural elements achieve good structural behaviors and should be a better way to amend partial performances of structural elements, such as lowering the self-weight, reducing the brittleness, improving the anti-cracking ability [12], and so on.

Lightweight aggregate concrete (LAC) with advantages of light self-weight, excellent thermal insulating properties, and outstanding seismic behavior is popularly used in long-span structures, bridges and so on [13,14]. However, it also has some shortcomings, such as high brittleness, the floating of lightweight aggregate during the vibrating process, and so on [15,16]. Previous studies suggest that the incorporation of rubber particles in concrete is a good way for reducing the brittleness of concrete [7], while self-compacting technology is a valid method to improve the maldistribution of aggregate in concrete [17]. In order to remedy the above deficiencies of LAC, self-compacting rubber lightweight aggregate concrete (SCRLC) is proposed on the basis of rubber lightweight aggregate concrete (RLAC) [18,19] and self-compacting lightweight aggregate concrete (SCLC) [20]. Lv et al. [21] points out that self-compacting technology can be successfully applied in RLAC through reasonable mix proportion design; meanwhile, SCRLC will achieve acceptable mechanical properties as the rubber particles substitution ratio is under 50%. Although a few of the research studies with respect to the properties of SCRLC have been carried out, very few existing research is reported about the application of SCRLC in structural elements so far. As a new structural material, applying it in structural elements and evaluating the structural performance is an essential path for popularization and application. In order to detect the feasibility and effectiveness of the utilization of SCRLC in structural elements, a composite slab is chosen in this research.

A composite slab comprised of profile steel sheeting and concrete is a popular form of floor slab utilized in a floor system of steel structure and composite structure [22]. Several advantages of composite slabs have been elaborated in previous studies [23,24,25,26,27], such as the decrease of self-weight, shortening of the construction period, simplification of the construction process, and so on. In this floor system, profiled steel sheeting can be used as a template for concrete pouring construction; meanwhile, it can be also used as a tension bar under loading. Thus, the template and tension bar will be saved. To date, numerous research studies have focused on the properties of composite slabs, and many mature design methods have been proposed for the guidance of engineering design [28,29,30,31,32,33]. Based on the virtues of SCRLC, instead of normal concrete (NC), SCRLC in composite slabs will acquire favorable effects; for example, waste tires will be recycled, the self-weight of composite slabs will be further reduced, the maldistribution of lightweight aggregate will be solved, the anti-cracking properties of composite slabs will be improved, and so on. However, there is very limited existing research on the structural properties of composite slabs comprised of SCRLC.

In this paper, a new type of composite slab composing of SCRLC and profile steel sheeting is prepared, and the flexural properties are investigated. The SCRLC mix proportions including six rubber particles substitution ratios from 0 to 50% are designed and two typically SCRLC mix proportions (0 and 30% in rubber particles substitution ratio) are selected to prepare composite slabs for experimental research. Each specimen has two shear spans (450 mm and 800 mm). The four typically composite slab specimens are tested to failure through four-point bending tests. The structural properties include failure modes, deflection at mid-span, profiled steel sheeting, and concrete surface stain at mid-span and end slippage. In addition, the finite element model is established to analyze the effect of SCRLC properties on the flexural behavior of a composite slab by ABAQUS software. The calculated method of the ultimate bending moment of composite slabs consisting of SCRLC is also proposed.

## 2. Experimental Program

### 2.1. Material Properties

#### 2.1.1. Concrete

**Figure 1:**
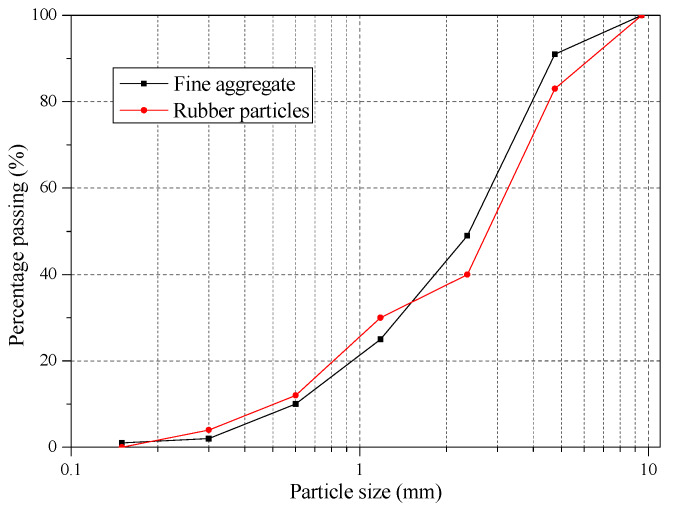
Particle size distribution of fine aggregate and rubber particles.

Six types of concrete were prepared in this investigation. The concrete was composed of ordinary Portland cement, fly ash, shale ceramsite (from 5 to 16 mm in size), sand, rubber particles (smaller than 4.75 mm in size), polycarboxylate-based high range water reducer, hydroxypropyl methylcellulose thickener, and water. The properties of raw materials could be found in Lv et al. [21]. The mix proportions are listed in Table 1. The rubber particles had the similar particle size distribution with sand and were utilized to replace sand by volume (shown in Figure 1). For the SCRLC30, the volume substitution ratio was 30%. The properties including the compressive strength, splitting tensile strength, elastic modulus, apparent density, and stress–strain curve were measured for each concrete batch. According to GB/T 50081 [34], the compressive strength and splitting tensile strength were measured on three cubic specimens of 100 mm × 100 mm × 100 mm, while the elastic modulus were measured on six prisms of 100 mm × 100 mm × 300 mm. The apparent density was determined following JGJ51-2002 [35]. The stress–strain curve was detected by a computer controlled electro-hydraulic servo universal testing machine at a constant rate loading displacement 0.002 mm/s. All samples were cured in a controlled environment of 20 ± 5 °C and relative humidity> 95% for 28 days. The flowability of SCRLC could be found in Lv et al. [21].

#### 2.1.2. Profiled Steel Sheeting

**Figure 2:**
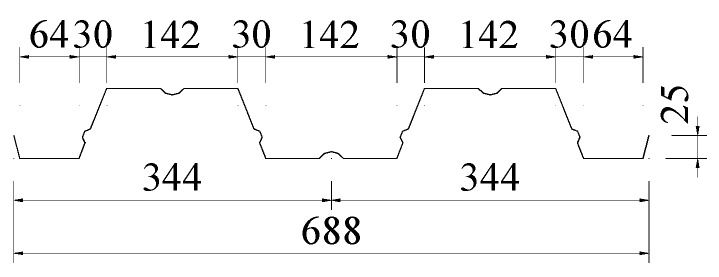
Cross-sectional dimensions of the profiled steel sheeting (all dimensions are in mm).

The profiled metal deck used in this study was YX76-344-688 profiled steel sheeting, and the model was DX51D + Z. The mechanical properties of profiled steel sheeting were elastic modulus 2.06 × 10^5^ MPa, yield strength 320 MPa, and tensile strength 405 MPa, respectively. The shape and size of profiled steel sheeting is shown in Figure 2, and the section properties are presented in Table 2. The thickness of the profiled steel sheeting was *t* = 1.2 mm.

#### 2.1.3. Reinforcement

The shear reinforcements used in the tests consisted of 8 or 14 numbers of 8 mm diameter reinforcing bars. The properties of reinforcing bars are shown in Table 3. In addition, one layer of steel mesh with 8 mm diameter and 180 mm spacing in both directions was installed at a distance of 25 mm from the upper surface of concrete. The shear reinforcement and steel mesh were screw thread bars and plain round bars, respectively.

### 2.2. Test Specimens

**Figure 3:**
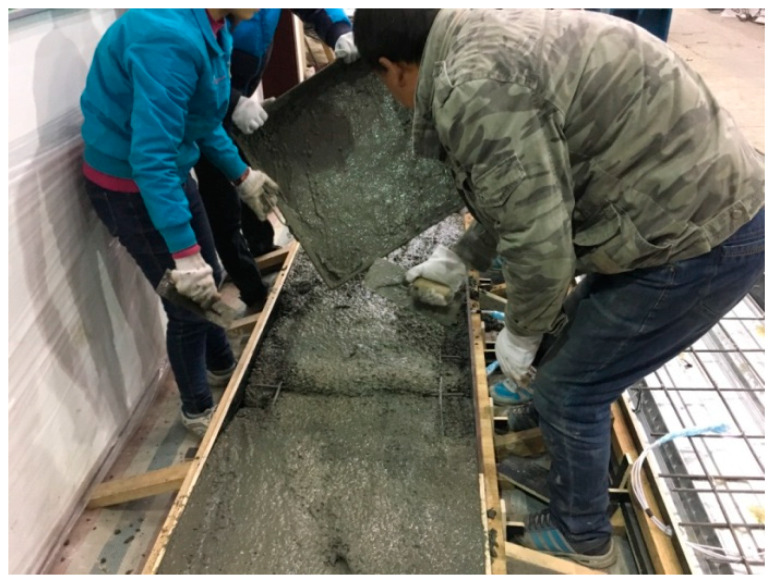
Manufacturing procedures of self-compacting rubber lightweight aggregate concrete (SCRLC) composite slab specimens.

Four composite slabs with two shear span and two types of SCRLC were manufactured, and the flexural performance was investigated through experimental research. The other types of SCRLC affecting the flexural behavior of composites slabs were analyzed by finite element software. Due to the excellent flowability of fresh concrete, the concrete was cast on the profiled steel sheeting without vibration. The manufacturing procedures of composite slabs is shown in Figure 3. After casting, the composite slabs were cured in a wet environment and a lab’s temperature for 28 days. Dimensions of the specimens are provided in Table 4.

**Figure 4:**
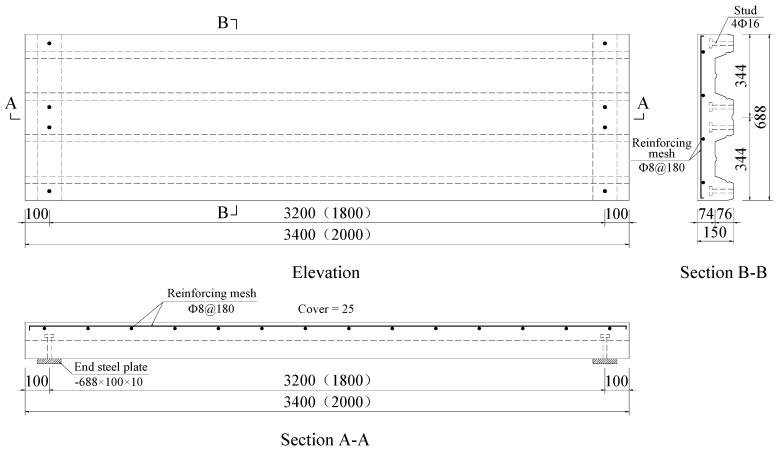
Details of test specimens (all dimensions are in mm). The symbol “Φ” represents the yield strength of the reinforcing bar is of grade 300 N/mm^2^.

Each specimen had a 100 mm overhang at both ends. Shear reinforcement with 8 mm diameter was arranged every 150 mm in the shear span along the length of composite slab and welded to the top flange of the profiled steel sheeting by argon arc welding. The steel mesh was configured at 25 mm above the surface of the composite slab to reduce the effect of shrinkage stress and temperature stress of SCRLC (shown in Figure 4). Four shear studs with 16 mm diameter were arranged at 100 mm from the both ends of the composite slab. In order to strengthen the connection of the shear stud and the profiled steel sheeting, headed shear studs were welded to a 10 mm thick steel plate underneath profiled steel sheeting at the support of each slab. The steel plate was used to replicate the steel beam, and each plate was 688 mm long by 100 mm wide. The details of the test specimens are shown in Figure 4.

### 2.3. Test Set-Up and Instrumentation

**Figure 5:**
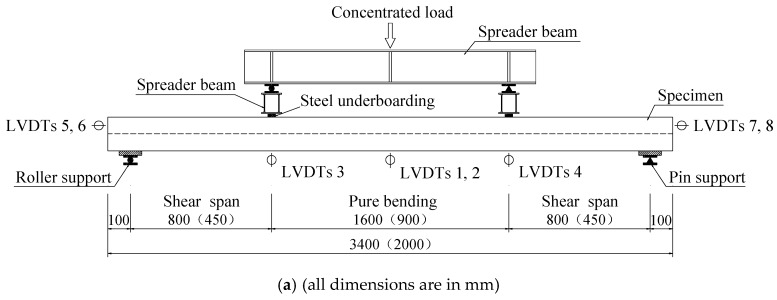

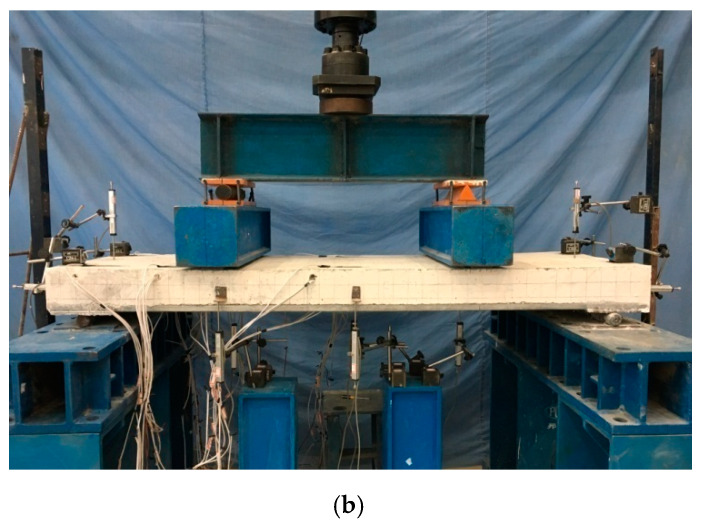
(**a**) Schematic diagram of test set-up and (**b**) Actual test set-up.

The spreader beam and loading arrangement is shown in Figure 5. A roller support and a pin support were supported at both ends of each slab. The load was linearly set up by a hydraulic jack of capacity 500 kN and applied in a force control manner. A spreader beam with two transverse beams was placed on the top surface of specimens to transfer loading.

**Figure 6:**
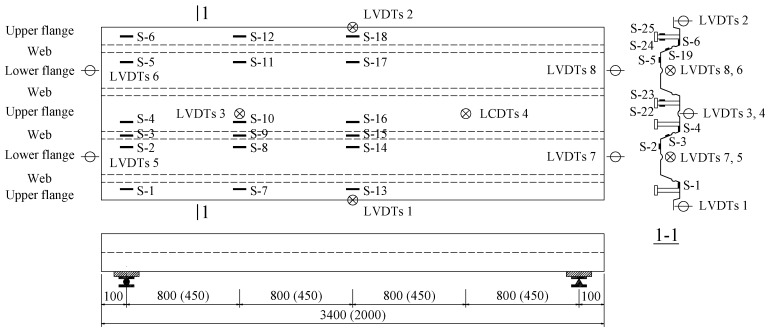
Location of linear variable displacement transducers (LVDTs) and strain gauges of profiled steel sheeting (all dimensions are in mm).

**Figure 7:**
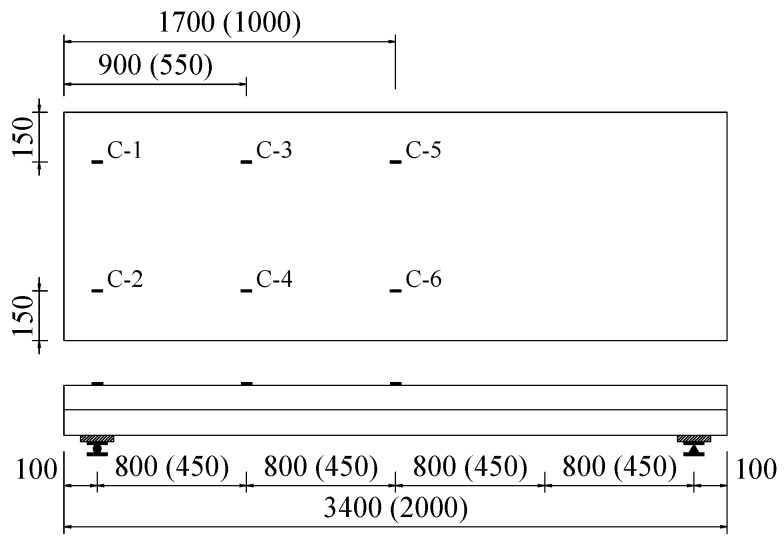
Location of strain gauges on the surface of concrete (all dimensions are in mm).

The instrumentations including a load cell (Shanghai Zhen-Dan Sensor Instrument Factory, Shanghai, China), linear variable displacement transducers (LVDTs, Jiangsu Donghua Testing Technology Co., ltd., Jingjiang, China), a hand-held microscope (Shanghai Deli stationery Co., ltd., Shanghai, China), and strain gauges (Xingtai Jinzhi Sensor Plant, Xingtai, China) were utilized in this test. The LVDTs were used to detect the deflection of slabs and end slip between the SCRLC and profiled steel sheeting at both ends of the slabs. The position of LVDTs can be seen in Figure 5 and Figure 6. Two types of strain gauges were used in this experimentation: one was used to detect the concrete strain on the top surface of the slab, while the other one was used to detect the strain of profiled steel sheeting at the upper flange, web, and lower flange of profiled steel sheeting. The position of strain gauges can be seen in Figure 6 and Figure 7. All strain gauges, LVDTs, and load cells were connected to a data acquisition system that stored the data during testing in a computer. The widths and locations of concrete cracks were straightly measured by a hand-held microscope with the accuracy of 0.01 mm when the loading was paused.

### 2.4. Loading Program

Before loading, pre-load was carried out to check whether the test instrumentations were normal and record the initial data. Loading was paused after every 5 kN (shear span 450 mm) or 10 kN (shear span 800 mm) increment and then we maintained the same load for 3 min. When the instrument data was stable, the new cracking was inspected, and existing crack widths and locations were measured. Data was recorded by an automatic data acquisition system continuously throughout the test. The test was terminated until the load declined under 15% of the ultimate values or the mid-span deflections reached to 1/25th of the span length.

## 3. Results and Discussion

### 3.1. Properties of SCRLC

The compressive strength, splitting tensile strength, elastic modulus, and apparent density of SCRLC with different rubber particles substitution ratio at 28 days are presented in Table 5. With the increasing rubber particles substitution ratio in SCRLC, the compressive strength, splitting tensile strength, elastic modulus, and apparent density of SCRLC dropped. Compared with the control mixture (SCLC), a reduction of around 54.4% for the compressive strength, 46.7% for the splitting tensile strength, 42.2% for the elastic modulus, and 14.2% for the apparent density occurred as the rubber particles substitution ratio was raised from 0% to 50%.

**Figure 8:**
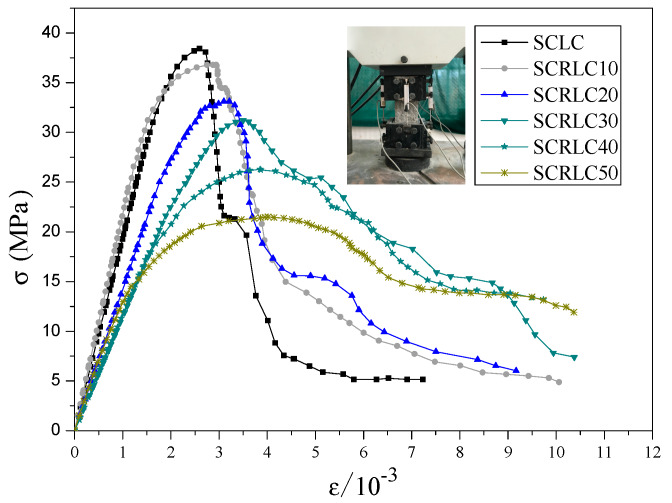
Whole curve of strain–stress under uniaxial compressive.

The whole curves of strain–stress under uniaxial compressive for SCRLC were obtained from experimental tests by Lv et al. [36], as shown in Figure 8. After analysis, the curve Equation (1) of strain–stress for SCRLC was given as follows:

(1) $$\frac{\sigma }{f_{p}}=\left\{ \begin{array}{l} \alpha \left(\frac{\varepsilon }{\varepsilon_{p}}\right)+(3-2\alpha ){\left(\frac{\varepsilon }{\varepsilon_{p}}\right)}^{2}+(\alpha -2){\left(\frac{\varepsilon }{\varepsilon_{p}}\right)}^{3}\;0\leq \left(\frac{\varepsilon }{\varepsilon_{p}}\right)\leq 1\\ \frac{\left(\frac{\varepsilon }{\varepsilon_{p}}\right)}{\beta {\left(\left(\frac{\varepsilon }{\varepsilon_{p}}\right)-1\right)}^{2}+\left(\frac{\varepsilon }{\varepsilon_{p}}\right)}\;1\leq \left(\frac{\varepsilon }{\varepsilon_{p}}\right) \end{array}\right.$$

where σ is the stress at any point of strain–stress curves under uniaxial compressive strength (MPa); *f_p_* is the peak stress (MPa); *ε* is the strain at any point of strain–stress curves under uniaxial compressive strength; *ε_p_* is the strain corresponding to the peak stress; and *α* and *β* are parameters regressing from strain–stress curves, which are shown in Table 6.

### 3.2. Experimental Study of Flexural Behavior of Composite Slabs

#### 3.2.1. Failure Procedure and Modes

**Figure 9:**
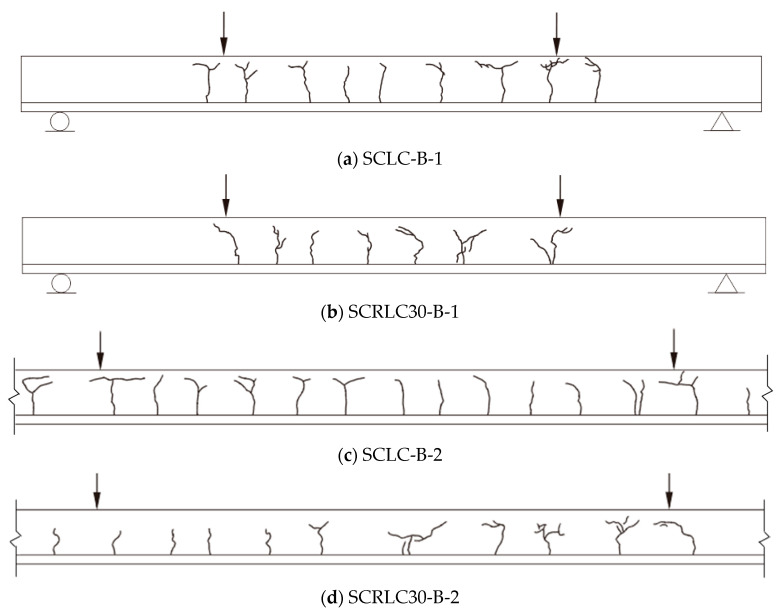
Final cracking patterns for each slab. (**a**) SCLC-B-1, (**b**) SCRLC30-B-1, (**c**) SCLC-B-2, (**d**) SCRLC30-B-2.

**Figure 10:**
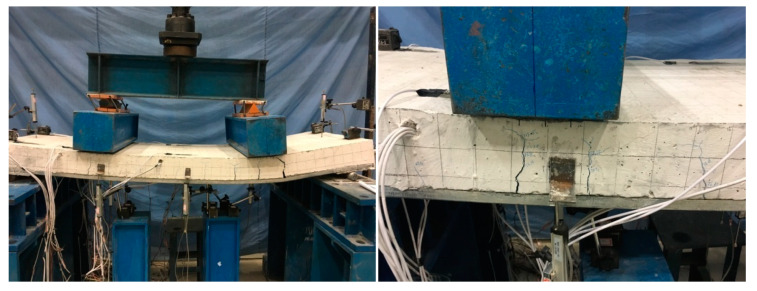
Typical failure modes of composite slab specimens (for SCLC-B-1).

The results detected from flexural experimental tests of composite slabs are shown in Table 7. *P_cr_* and *u_cr_* were the load and deflection when the initial cracking appeared in concrete. *P_y_* and *u_y_* were the load and deflection when the composite slabs yielded. *P_u_* and *u_u_* were the load and deflection when the composite slabs reached the ultimate load capacity. The final cracking patterns for each slab are exhibited in Figure 9. The typical failure modes of composite slab specimens (for SCRLC30-B-1) are shown in Figure 10.

Specimens SCLC-B-1 and SCLC-B-2 exhibited an almost alike failure pattern. Meanwhile, specimens SCRLC30-B-1 and SCRLC30-B-2 had the similar failure mode. For all four composite slabs, at the initial stage of loading, the concrete and profiled steel sheeting worked together well. As the applied load increased, the deflection of the mid-span slabs raised. When the load was near the cracking load, a cracking noise was heard, and little fine cracks were noticed on the bottom of the concrete surface at the loading points. With the continuing increase of applied load, the existing cracks extended upward and widened, and new cracks appeared constantly near the loading points and the constant moment region of the slabs. At the final failure stage, compared with specimens SCRLC30-B-1 and SCRLC30-B-2, specimens SCLC-B-1 and SCLC-B-2 had more cracks and the width of most cracks was much bigger, although the cracks had not stretched to full depth near the loading points. It indicated that the utilization of SCRLC30 in composite slabs would improve the crack resistance property of composite slabs. The end-slip of four composite slabs were all very little. The failure mode of four composite slabs were all typical flexural failure.

#### 3.2.2. Load–Deflection Curves

**Figure 11:**
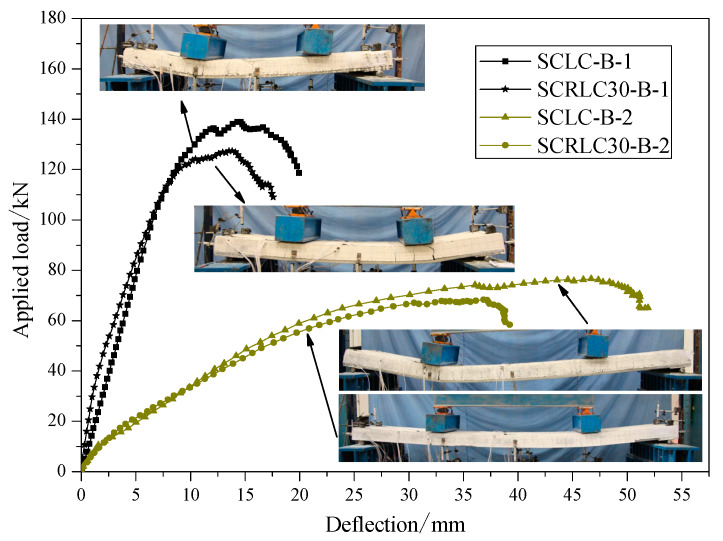
Load–deflection curves of different composite slabs at mid-span.

The load–deflection curves of four composite slabs at mid-span are presented in Figure 11. The same shear span specimens had similar load–deflection curves. Compared with shear span 450 mm specimens, shear span 800 mm specimens had a lower ultimate load and higher mid-span deflection. For each specimen, obviously, a linear increase in deflection occurred as the applied loading was raised before the composite slabs yielded. Despite the cracks appearing on the surface of the concrete, the concrete and profiled steel sheeting still worked well together. The ultimate load and corresponding deflection of composite slabs consisting of SCRLC30 were inferior to the composite slabs consisting of SCLC regardless of short shear span and long shear span. It might be mainly attributed to the reduction of concrete strength. The compressive strength of concrete decreased from 45.6 to 33.8 MPa, which was about a 25.9% reduction ratio as the rubber particles substitution ratio increased from 0% to 30%, the variation of the ultimate load of the shear span for the 800 mm and shear span 450 mm composite slabs was from 139.4 to 128.9 kN and 76.7 to 68.7 kN, which were 7.5% and 10.4% reduction ratios, respectively. The final deflection of the composite slab consisting of SCRLC30 was less than the composite slabs consisting of SCLC, no matter whether they were the shear span 450 mm specimens or shear span 800 mm specimens.

The laboratorial initial cracking load to the ultimate load ratios of four composite slabs were calculated and summarized in Table 7. For the same shear span specimens, compared with composite slabs consisting of SCLC, the composite slabs consisting of SCRLC30 had greater initial cracking load to ultimate load ratios. It meant that the anti-cracking ability under the loading of composite slabs would be significantly improved as SCRLC30 substituted SCLC in composite slabs.

#### 3.2.3. Profiled Steel Strain and Concrete Surface Strain Analysis

**Figure 12:**
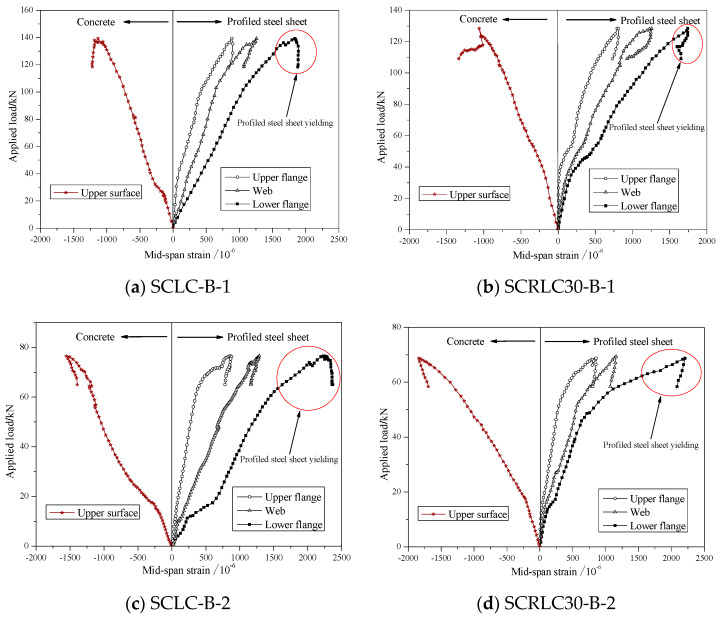
Load-profiled steel sheeting/concrete strain curves of different composite slabs at mid-span. (**a**) SCLC-B-1, (**b**) SCRLC30-B-1, (**c**) SCLC-B-2, (**d**) SCRLC30-B-2.

The load-profiled steel sheeting/concrete strain curves of different composite slabs at mid-span are shown in Figure 12. For concrete, the strains at the upper surface of composite slab were negative for all four composite slabs. It indicated all the concrete at the upper surface was in press condition. Before the ultimate load, the load increased approximately linearly with the increase of strain on the upper surface of composite slabs. The peak strain for each composite slabs was lower than the ultimate compressive strain of concrete (SCLC: 2.6471 × 10^−3^; SCRLC30: 3.5011 × 10^−3^). It meant that the concrete should be intact at the upper surface. This conclusion was also verified by the testing phenomenon.

For profiled steel sheeting, the strains at the upper flange, web, and lower flange were all positive. The entire profiled steel sheeting was in a state of tension for all four composite slabs. In the initial stage, the load increased by the strain nearly linearly. With the continuing increase in the applied load, the strain at the bottom flange of the profiled steel sheeting exceeded the yield strain (1553 × 10^−6^) and yielded. Whereas, the strain at the upper flange and web of the profiled steel sheeting was always lower than the yield strain (1553 × 10^−6^) during the loading process. The upper flange and web of the profiled steel sheeting was still in the elastic range until the test ended. Shear span 800 mm specimens had greater strain than shear span 450 mm specimens.

**Figure 13:**
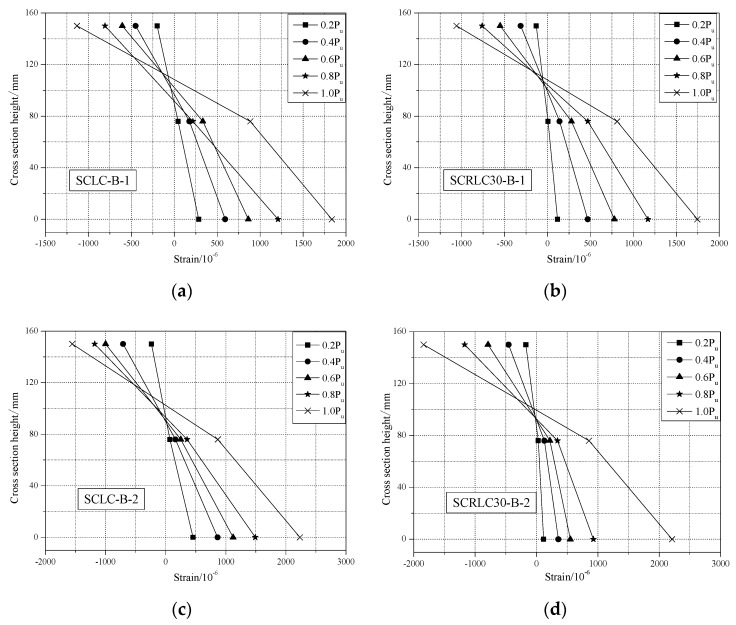
Sectional strain distribution at mid-span for different composite slab specimens. (**a**) SCLC-B-1, (**b**) SCRLC30-B-1, (**c**) SCLC-B-2, (**d**) SCRLC30-B-2.

The section strain distribution at the mid-span for different composites slabs is presented in Figure 13. It can be seen that the variation of strain along the height of the cross-section at the upper surface of concrete, upper flange, and lower flange of profiled steel sheeting were approximately linear as the applied load increased. The deformation of SCLC slabs and SCRLC30 slabs under the applied load obeyed the plain section assumption.

#### 3.2.4. Load–Slip Curves

**Figure 14:**
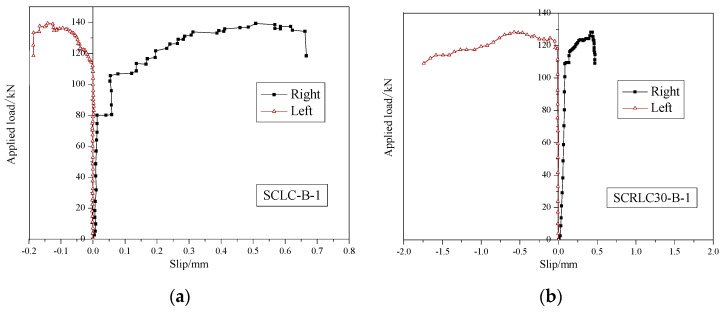

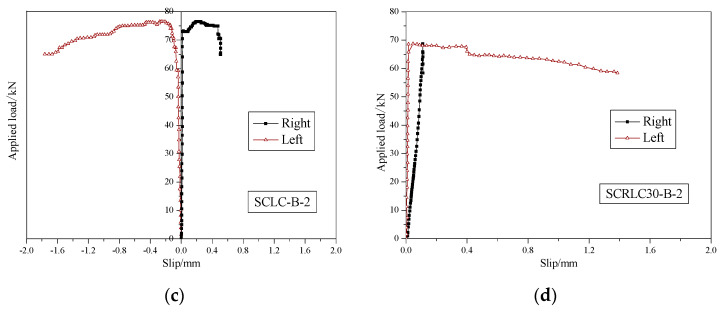
Load–slip behavior of different composites slabs. (**a**) SCLC-B-1, (**b**) SCRLC30-B-1, (**c**) SCLC-B-2, (**d**) SCRLC30-B-2.

**Figure 15:**
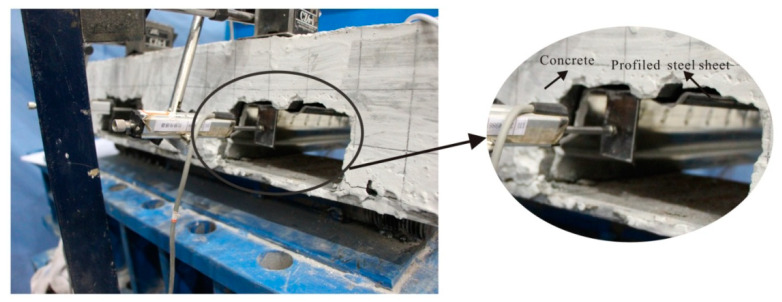
Typical final end slip between concrete and profiled steel sheeting (for an SCLC-B-2 slab).

Figure 14 exhibited the end load–slip behavior of different composites slabs. The load–slip curves showed a slight or no slip between the concrete and profiled steel sheeting at the initial loading stage. With the increase in load, the slippage augmented; in particular, the load exceeded the peak load. At the peak load stage, the slippage of four composite slabs were still keeping a lower level, the maximum slippage was 0.58 mm, which occurred in the SCLC-B-1 slab (as seen in Figure 15). The relatively small slippage was mainly according to the use of shear stud and shear bars. It indicated that the failure form of composite slabs was typical bending failure.

### 3.3. Finite Element Analysis of Flexural Behavior of Composite Slabs

In this research, ABAQUS software was utilized to study the further investigation on the flexural behavior of SCRLC composite slabs.

#### 3.3.1. General Structural Model

**Figure 16:**
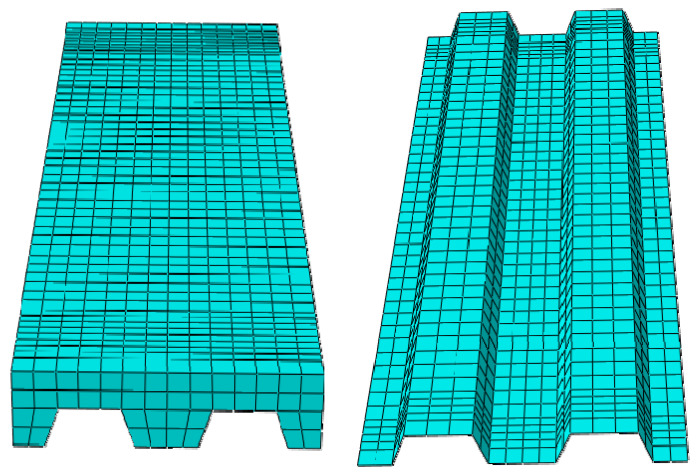
Finite element model.

A 3D finite element model was established to account for the geometric and material nonlinearities in composite slabs. The concrete was modeled using the “8-node linear brick, reduced integration elements (C3D8R)”. The concrete performance parameters used in the finite element model were determined by materials experiments and shown in Section 3.1. The profiled steel sheeting used in the finite element model was modeled using the “4-node doubly curved shell, reduced integration elements (S4R)”. The material properties are shown in Section 2.1.2. An elastic–plastic model based on Von Mises yield criterion and kinematic hardening criterion was utilized to treat the plasticity of profiled steel sheeting. Due to small slippage between the concrete and profiled steel sheeting, a tie contact was chosen to handle the interaction effect between the concrete and profiled steel sheeting. In view of the accuracy and efficiency of finite element analysis (FEA), the global element sizes were limited to 50 mm for concrete and profiled steel sheeting and 25 mm for two supports. The finite element model of a simulated experimental composites slab is presented in Figure 16.

**Figure 17:**
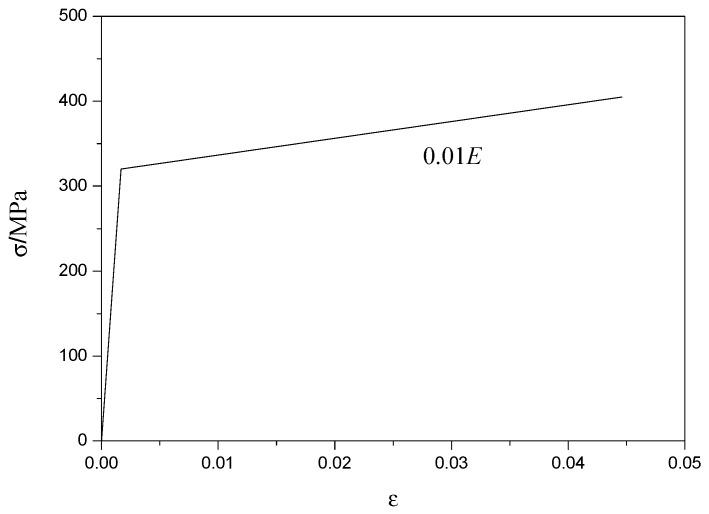
Stress–strain relationship of profiled steel sheeting.

The double broken line model was used to model the profiled steel sheeting, as shown in Figure 17. The elastic module of profiled steel sheeting during the elastic stage was *E* = 2.06 × 10^5^ N/mm^2^, while the elastic module of the profiled steel sheeting during the strengthening stage was *E*’ = 0.01*E*. The “concrete damaged plasticity” mechanical model in ABAQUS was used to model SCRLC. The compressive behavior of SCRLC was simulated by the compressive stress–strain relationship, as shown in Equation (1). The tensile behavior of SCRLC was simulated in accordance with the Chinese Code for the design of concrete structures (GB50010 2010), as shown in the following Equation (2).

(2) $$\frac{\sigma }{f_{t}}=\left\{ \begin{array}{ll} 1.2\frac{\varepsilon }{\varepsilon_{p}}-0.2{\left(\frac{\varepsilon }{\varepsilon_{p}}\right)}^{6} & 0\leq \frac{\varepsilon }{\varepsilon_{p}}\leq 1\\ \frac{\left(\frac{\varepsilon }{\varepsilon_{p}}\right)}{\alpha_{t}{\left(\frac{\varepsilon }{\varepsilon_{p}}-1\right)}^{1.7}+\frac{\varepsilon }{\varepsilon_{p}}} & \frac{\varepsilon }{\varepsilon_{p}}\geq 1 \end{array}\right.$$

where *α_t_* = 0.312*f_t_*^2^, *f_t_* was the tensile strength of SCRLC, which could be seen in Table 5.

The supports were constrained by two translational degrees of freedom, namely, *u_y_* = *u_z_* = 0. For the purpose of simulating the load distribution types for the composite slabs, the loading beam was coupled with a loading point. In this way, the loading point and loading beam had the same vertical displacement. The loading method applied in FEM was according to the experimental loading procedure.

#### 3.3.2. Verification of Finite Element Model

**Figure 18:**
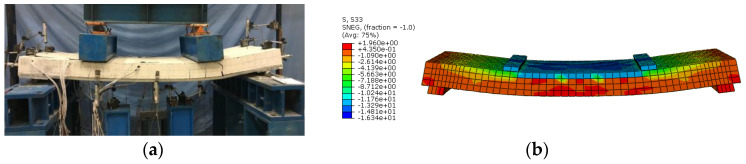
Comparison of failure modes for specimens (for SCRLC30-B-1 slabs): (**a**) test failure; (**b**) FEA failure.

For verifying the accuracy and effectiveness of the finite element model, the numerical results were contrasted with experimental results. Figure 18 exhibited the failure modes of test and FEA for composite slabs. It can be seen from Figure 18b that the upper composite slab was in compression while the lower composite slab was in tension, which was in line with the experimental results. The FEA failure mode was also quite similar to the test failure.

**Figure 19:**
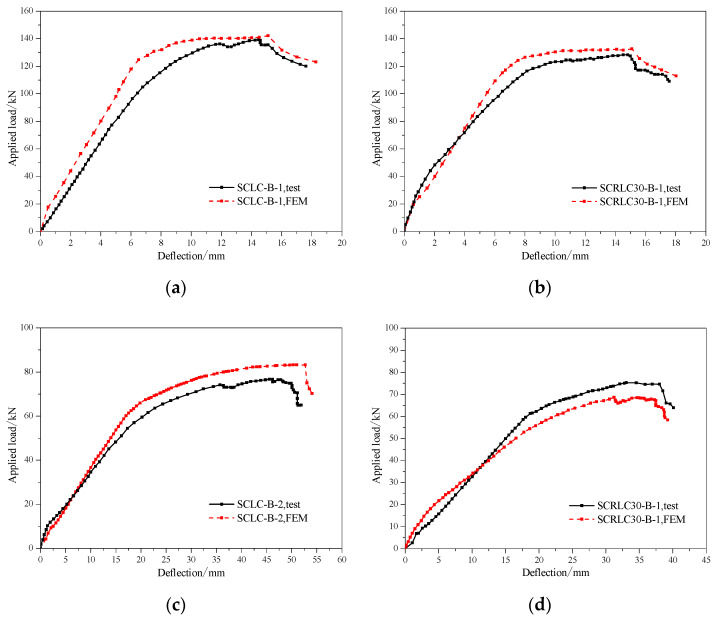
Comparison of load–deflection curves of composite slabs at mid-span between tests and FEA. (**a**) SCLC-B-1, (**b**) SCRLC30-B-1, (**c**) SCLC-B-2, (**d**) SCRLC30-B-2.

The comparison of load–deflection curves of composite slabs at mid-span between tests between experimental results and FEA are presented in Figure 19. It was observed that the load–deflection curves of composite slabs at the mid-span between tests and FEA had a similar trend. From Table 8, it is evident that the ratios of numerical results to testing results were between 1.04 and 1.09 for the yield load, 0.92 and 1.02 for yield deflection, 1.02 and 1.08 for the peak load, and 1.02 and 1.05 for peak deflection. To sum up, the finite element model could simulate the test process well and could be utilized to conduct the following parametric research studies on the flexural properties of composite slabs.

#### 3.3.3. Parametric Studies on Flexural Properties of Composite Slabs

**Figure 20:**
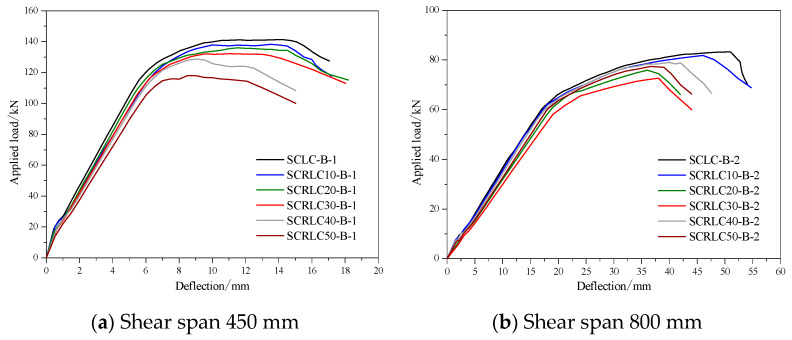
Load–deflection curves of different composite slabs at mid-span by FEA. (**a**) Shear span 450 mm, (**b**) Shear span 800 mm.

A series of parametric studies with a variation of SCRLC strength on the flexural behaviors of composite slabs were carried out by FEA. The load–deflection curves of different composite slabs at mid-span with a shear span of 450 mm and a shear span of 800 mm are shown in Figure 20. The numerical results containing yield load, ultimate load, and deflection corresponding to yield load and ultimate load are summarized in Table 9. With the increase of the rubber particles substitution ratio in SCRLC, the yield load, ultimate load, and deflection corresponding to the yield load and ultimate load of composite slabs decreased. As the compression strength of SCRLC dropped from 45.6 to 20.8 MPa with 54.4% reduction, the ultimate load of composite slabs reduced from 142.2 to 118.1 kN with 16.9% reduction for short shear span and 82.8 to 71.7 kN with 13.4% reduction for long shear span. Obviously, the reduction of concrete strength was much more than the reduction of the bearing capacity of composite slabs. It indicated that concrete strength had less influence on the flexural bearing capacity of composite slabs.

Comparing with NC composite slabs [22], SCRLC composite slabs had the similar ultimate load when the properties of concrete, profile steel sheeting, and the size of composite slabs were approximate. Whereas, due to the low apparent density and favorable damping capacity of SCRLC [37], compared with NC composites slabs, the self-weight of SCRLC composites slabs will reduce by about 30% and the seismic capacity of composites slabs will be improved.

### 3.4. Evaluation of Flexural Capacity of Composite Slabs

**Figure 21:**
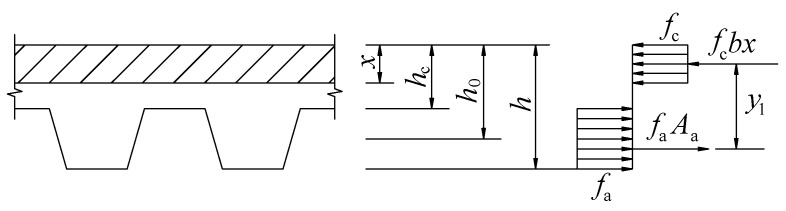
Calculating diagram of flexural capacity of composite slabs.

According to JGJ138-2016 [38], the calculating diagram of the flexural capacity of composite slabs is shown in Figure 21, while the ultimate bending moment of composite slabs could be calculated by Equation (3).

(3) $$M_{u}=f_{c}bx\left(h_{0}-\frac{x}{2}\right)$$

The height of compressive zone of composite slabs at ultimate state *x* can be expressed as:

(4) $$x=\frac{A_{a}f_{a}}{f_{c}b}$$

where *M_u_* is the cross-section ultimate bending moment of the composite slab, *b* is the calculative width of a composite slab section, *h_c_* is the distance between the upper surface of concrete and the upper flange of profiled steel sheeting, *h*_0_ is the effective height of the composite slab, *h* is the thickness of the composite slab, *f_a_* is the design value of tensile strength of profiled steel sheeting, *f_c_* is the design value of compressive strength of concrete, and *A_a_* is the cross-sectional area of profiled steel sheeting.

On the basis of the Equations (3) and (4), the calculated value of the ultimate bending moment of composite slabs and the ratio of calculated values to experimental values are listed in Table 10. For all 12 composite slabs, the calculated values of the ultimate bending moment were greater than the experimental values. The ratio of calculated values to experimental values of the ultimate bending moment of composite slabs was between 1.15 and 1.35. It meant that using Equations (3) and (4) to predict the flexural capacity of SCRLC composite slabs was unsuitable. It might be mainly due to the inaccurate assumption in Equations (3) and (4). Equations (3) and (4) considered that the profiled steel sheeting had the same stress at the upper flange, web, and lower flange when the composite slabs reached their ultimate carrying capacity. However, the experimental and numerical results indicated that was not the case. The strain of the upper flange of the profiled steel sheet detected by experiment and FEA is summarized in Table 11. The ratio of the strain of the upper flange to the yield strain of the profiled steel sheeting ranged from 0.52 to 0.68, with an average value of 0.60. It showed that the strain of the upper flange of the profiled steel sheeting could only reach approximately 60% of the yield strain of the profiled steel sheeting when the composite slabs reached their ultimate flexural carrying capacity. Hence, modifying the traditional calculating method of the flexural capacity of composite slabs was necessary to ensure the calculated value of the ultimate bending moment of SCRLC composite slabs, which was in keeping with the experimental result.

Based on the above analysis, in order to established an effective, reliable, and simple calculated method to evaluate the flexural capacity of SCRLC composite slabs, the following assumptions were made:

(1) The bending deflection of SCRLC composite slabs was accordance with the plane section assumption;
(2) The tensile strength of SCRLC in the tensile zone of composite slabs was ignored;
(3) The stress of the upper flange of profiled steel sheeting was 60% of the yield stress of profiled steel sheeting when the composite slabs reached their ultimate flexural carrying capacity.

**Figure 22:**
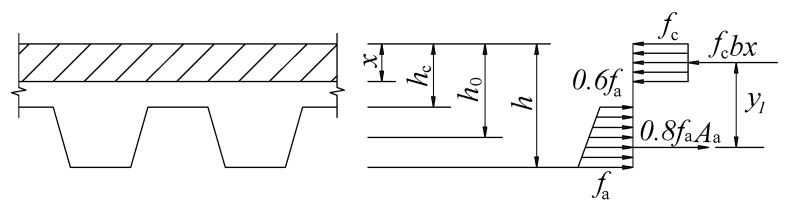
Modified calculating diagram of flexural capacity of composite slabs.

The modified calculating diagram of the flexural capacity of SCRLC composite slabs is presented in Figure 22.

According to the force balance of the section of composite slabs, the modified height of the compressive zone of composite slabs at ultimate state *x*’ can be illustrated as:

(5) $$(\frac{1+0.6}{2})A_{a}f_{a}=f_{c}bx^{\prime }.$$

The modified calculated equation of the ultimate bending moment of SCRLC composite slabs *M’_u_* was expressed as:

(6) $${M^{\prime }}_{u}=f_{c}bx^{\prime }\left(h_{0}-\frac{x^{\prime }}{2}\right)=0.8A_{a}f_{a}x^{\prime }\left(h_{0}-\frac{x^{\prime }}{2}\right).$$

Table 12 showed the experimental and modified calculated value of the ultimate bending moment of composite slabs. It could be seen that most of the modified calculated values of the ultimate bending moment of the composite slabs were in good aggregate with the experimental value. The average value of the ratio of the modified calculated value to the experimental value was 1.00 with a standard deviation of 0.04. It meant that the modified calculated method was suitable for predicting the flexural capacity of SCRLC composite slabs.

For keeping the calculated method of the ultimate bending moment of SCRLC composite slabs consistent with the Chinese Industry Standard JGJ138-2016 (China, 2016), the modified calculated equation could be also expressed as:

(7) $$M_{u}^{\prime }=0.81M_{u}=0.81f_{c}bx\left(h_{0}-\frac{x}{2}\right).$$

## 4. Conclusions

Experimental research and the FEA of the flexural properties of SCRLC composite slabs were carried out in this paper. Based on the experimental and analytical results, the main conclusions are drawn as following:

(1) Self-compacting technology was successfully used in the preparation of rubber lightweight aggregate concrete to make composite slabs. Four composite slabs with different shear spans (450 mm and 800 mm) and SCRLC (0 and 30% in rubber particles substitution ratio) were prepared, and the flexural strength tests were conducted. Comparing with SCLC composite slabs, SCRLC30 composite slabs had better anti-cracking ability under loading.
(2) An accurate and effective finite element model was established to account for further understanding on the effect of SCRLC strength on the flexural properties of composite slabs. With the increase of rubber particles substitution ratio in SCRLC, the yield load, ultimate load, and deflection corresponding to the yield load and ultimate load of composite slabs decreased. The reduction of SCRLC strength was much more than the reduction of the bearing capacity of corresponding composite slabs.
(3) Comparing with NC composite slabs, SCRLC composite slabs had the similar ultimate load when the properties of the concrete, profile steel sheeting, and size of the composite slab were approximate. However, the self-weight of the SCRLC composites slabs reduced by about 30%. Applying SCRLC in composites slabs was feasible and would be beneficial to improve the anti-cracking ability of composites slabs.
(4) The traditional calculated method of the flexural bearing capacity of NC composite slabs was unsuitable for analyzing the flexural bearing capacity of SCRLC composite slabs. After analysis, a modified calculated method was proposed for the evaluation of the flexural capacity of SCRLC composite slabs.

In summary, SCRLC is a new type of structural material with excellent physical and mechanical properties. This research is a beneficial attempt to utilize SCRLC in structural members. The results will provide guidance on the design and usage of SCRLC composite slabs and unavoidably bring about huge environmental and societal benefits.

## Figures and Tables

**Table 1:** Mix proportions for concrete.

Type of Concrete	Substitution (by Volume)	Weight Per Cubic Meter (kg/m^3^)							
		Cement	Fly Ash	Rubber Particles	Sand	Shale Ceramsite	Thickener	Water Reducer	Water
SCLC	0	425	85	0	700	610	0.204	5.1	179
SCRLC10	10%	425	85	31	630	610	0.204	5.1	179
SCRLC20	20%	425	85	62	560	610	0.204	5.1	179
SCRLC30	30%	425	85	93	490	610	0.204	5.1	179
SCRLC40	40%	425	85	124	420	610	0.204	5.1	179
SCRLC50	50%	425	85	155	350	610	0.204	5.1	179

**Table 2:** Section properties of profiled steel sheeting.

Type	Weight (kg/m^2^)	Moment of Inertia, *I* (cm^4^/m)	Sectional Resistance Moment, *W* (cm^3^/m)
YX76-344-688	14.10	176.49	44.12

**Table 3:** Properties of reinforcing bars.

Diameter (mm)	Yield Strength( MPa)	Tensile Strength (MPa)	Elastic Modulus (GPa)	Elongation (%)
8	335	450	206	25

**Table 4:** Dimensions of composite slab specimens.

Slab Specimen	Length, *L* (mm)	Width, *B* (mm)	Thickness of Slab, *d*_s_ (mm)	Shear Span, *L*_x_ (mm)	Substitution (by Volume) (%)
SCLC-B-1	2000	688	150	450	0
SCLC-B-2	3400	688	150	800	
SCRLC30-B-1	2000	688	150	450	30
SCRLC30-B-2	3400	688	150	800	

**Table 5:** Properties of SCRLC.

Type of Concrete	Compressive Strength, (MPa)	Elastic Modulus, (GPa)	Splitting Tensile Strength (MPa)	Apparent Density (kg/m^3^)
SCLC	45.6	26.8	4.41	1921
SCRLC10	43.3	24.3	4.19	1867
SCRLC20	39.4	22.7	3.82	1825
SCRLC 30	33.8	19.9	3.36	1765
SCRLC40	25.3	18.1	2.77	1709
SCRLC50	20.8	15.5	2.35	1648

**Table 6:** Parameters *α* and *β* for strain–stress curves of self-compacting rubber lightweight aggregate concrete (SCRLC).

Parameter	SCLC	SCRLC10	SCRLC20	SCRLC30	SCRLC40	SCRLC50
*α*	1.84	1.94	2.13	2.35	2.51	2.89
*β*	16	8	6	1.6	1.4	1.2

**Table 7:** The results from experimental tests.

Symbol of Specimen	Initial Cracking State of Concrete		Yielding State of Slabs		Ultimate Load Capacity Status of Slabs		*P_cr_*/*P_u_*
	*P_cr_*/(kN)	*u_cr_*/(mm)	*P_y_*/(kN)	*u_y_*/(mm)	*P_u_*/(kN)	*u_u_*/(mm)	
SCLC-B-1	35.5	2.23	123.5	9.41	139.4	14.48	0.232
SCLC-B-2	11.1	1.36	70.0	25.93	76.7	46.21	0.144
SCRLC30-B-1	39.2	1.45	108.6	7.21	128.9	14.81	0.304
SCRLC30-B-2	12.4	2.24	52.2	18.18	68.7	36.79	0.181

**Table 8:** Comparison of test and FEA results.

Symbol of Specimen	*P_y_* (kN)			*u_y_* (mm)			*P_u_* (kN)			*u_u_* (mm)		
	Test	FEM	FEM/Test	Test	FEM	FEM/Test	Test	FEM	FEM/Test	Test	FEM	FEM/Test
SCLC-B-1	123.5	135.2	1.09	9.4	8.6	0.92	139.4	142.2	1.02	14.5	15.0	1.03
SCLC-B-2	108.6	117.3	1.08	7.2	6.7	0.93	128.9	132.6	1.03	14.8	15.1	1.02
SCRLC30-B-1	70.0	72.8	1.04	25.9	26.3	1.02	76.7	82.8	1.08	46.2	48.6	1.05
SCRLC30-B-2	52.2	56.9	1.09	18.2	18.6	1.02	68.7	74.5	1.08	36.8	38.0	1.02

**Table 9:** Analysis results of FEA.

Symbol of Specimen	Yielding State of Slabs		Ultimate Load Capacity Status of Slabs		Substitution (by Volume) (%)
	*P_y_*/(kN)	*u_y_*/(mm)	*P_u_*/(kN)	*u_u_*/(mm)	
SCLC-B-1	135.2	8.6	142.2	15.0	0
SCLC-B-2	72.8	25.9	82.8	48.6	
SCRLC10-B-1	124.3	7.0	138.3	13.5	10%
SCRLC10-B-2	68.2	22.8	81.7	46.0	
SCRLC20-B-1	122.3	6.6	135.4	12.5	20%
SCRLC20-B-2	65.5	20.7	79.3	40.4	
SCRLC30-B-1	117.3	6.7	132.6	15.1	30%
SCRLC30-B-2	56.9	18.6	74.5	38.0	
SCRLC40-B-1	118.8	6.7	128.9	9.5	40%
SCRLC40-B-2	63.2	20.8	75.9	36.0	
SCRLC50-B-1	110.2	6.6	118.1	9.0	50%
SCRLC50-B-2	61.8	20.5	71.7	36.2	

**Table 10:** Comparison between experimental and calculated values of the ultimate bending moment of composite slabs.

Symbol of Specimen	Calculated Value *M_u_*/kN·m	Experimental Value *M*/kN·m	*M_u_*/*M*
SCLC-B-1	38.17	32.0	1.19
SCLC-B-2	38.17	33.1	1.15
SCRLC10-B-1	38.09	31.1	1.22
SCRLC10-B-2	38.09	32.7	1.16
SCRLC20-B-1	37.69	30.5	1.24
SCRLC20-B-2	37.69	31.7	1.19
SCRLC30-B-1	37.50	29.8	1.26
SCRLC30-B-2	37.50	29.8	1.26
SCRLC40-B-1	36.55	29.0	1.26
SCRLC40-B-2	36.55	30.4	1.20
SCRLC50-B-1	35.62	26.6	1.34
SCRLC50-B-2	35.62	28.9	1.23

**Table 11:** Strain of profiled steel sheet.

Symbol of Specimen	Upper Flange *ε_u_* (×10^−6^)		Yielding Strain *ε_y_* (×10^−6^)	*ε_u_*/*ε_y_*
	Numerical Results	Experimental Results		
SCLC-B-1	972	898	1553	0.62 (0.58)
SCLC-B-2	942	811	1553	0.61 (0.52)
SCRLC10-B-1	908	/	1553	0.58
SCRLC10-B-2	910	/	1553	0.59
SCRLC20-B-1	814	/	1553	0.52
SCRLC20-B-2	898	/	1553	0.58
SCRLC30-B-1	915	879	1553	0.59 (0.57)
SCRLC30-B-2	984	857	1553	0.63 (0.55)
SCRLC40-B-1	781	/	1553	0.50
SCRLC40-B-2	1063	/	1553	0.68
SCRLC50-B-1	961	/	1553	0.62
SCRLC50-B-2	990	/	1553	0.64
Average value	—	—	—	0.60
Standard deviation	—	—	—	0.047

**Table 12:** Comparison between experimental and modified calculated value of the bending moment of composite slabs.

Symbol of Specimen	Modified Calculated Value *M’_u_*/kN·m	Experimental Value *M*/kN·m	*M’_u_*/*M*	*M’_u_*/*M_u_*
SCLC-B-1	30.95	32.0	0.97	0.81
SCLC-B-2	30.95	33.1	0.94	0.81
SCRLC10-B-1	30.89	31.1	0.99	0.81
SCRLC10-B-2	30.89	32.7	0.94	0.81
SCRLC20-B-1	30.64	30.5	1.00	0.81
SCRLC20-B-2	30.64	31.7	0.97	0.81
SCRLC30-B-1	30.52	29.8	1.02	0.81
SCRLC30-B-2	30.52	29.8	1.02	0.81
SCRLC40-B-1	29.91	29	1.03	0.82
SCRLC40-B-2	29.91	30.4	0.98	0.82
SCRLC50-B-1	29.31	26.6	1.10	0.82
SCRLC50-B-2	29.31	28.9	1.01	0.82
Average value	—	—	1.00	0.81
Standard deviation	—	—	0.04	0.004
